# Pretreatment Inflammatory-Nutritional Biomarkers Predict Responses to Neoadjuvant Chemoradiotherapy and Survival in Locally Advanced Rectal Cancer

**DOI:** 10.3389/fonc.2021.639909

**Published:** 2021-03-17

**Authors:** Yijun Wang, Lejun Chen, Biyun Zhang, Wei Song, Guowei Zhou, Ling Xie, Dahai Yu

**Affiliations:** ^1^Department of Radiation Oncology, Jiangsu Province Hospital of Chinese Medicine, Affiliated Hospital of Nanjing University of Chinese Medicine, Nanjing, China; ^2^Department of General Surgery, Jiangsu Province Hospital of Chinese Medicine, Affiliated Hospital of Nanjing University of Chinese Medicine, Nanjing, China; ^3^Department of Pathology, Jiangsu Province Hospital of Chinese Medicine, Affiliated Hospital of Nanjing University of Chinese Medicine, Nanjing, China

**Keywords:** rectal cancer, prognostic nutritional index, systemic inflammatory response, pathological response, survival

## Abstract

**Background:**

To evaluate the value of pretreatment inflammatory-nutritional biomarkers in predicting responses to neoadjuvant chemoradiotherapy (nCRT) and survival in patients with locally advanced rectal cancer (LARC).

**Methods:**

Patients with LARC who underwent nCRT and subsequent surgery between October 2012 and December 2019 were considered for inclusion. Neutrophil to lymphocyte ratio (NLR), platelet to lymphocyte ratio (PLR), lymphocyte to monocyte ratio (LMR), and prognostic nutritional index (PNI) were calculated from according to routine laboratory data within 1 week prior to nCRT. The correlations between baseline inflammatory-nutritional biomarkers and responses were analyzed using Chi-square test or Fisher’s exact test, and multivariate logistic regression analysis was performed to identify the independent predictors of pathological responses to nCRT. Univariate and multivariate Cox proportional hazard models were used to assess the correlations of predictors with disease-free survival (DFS) and overall survival (OS).

**Results:**

A total of 273 patients with LARC were enrolled in this study. Higher LMR and PNI were observed in the good-response group, meanwhile higher NLR and PLR were observed in the poor-response group. Multivariate logistic regression analysis results revealed that PLR and PNI independently predicted responses to nCRT. Multivariable Cox regression analysis determined that PNI was an independent predictor of DFS and OS in patients with LARC. The value of pretreatment PNI in predicting responses and survival was continuously superior to those of NLR, PLR, and LMR. The optimal cutoff value of the PNI was approximate 45. Subgroup analyses indicated that the pathological responses and survival in the high PNI group (≥ 45) were significantly better than those in the low PNI group (< 45), especially in patients with clinical stage III rectal cancer.

**Conclusion:**

The pretreatment PNI can serve as a promising predictor of response to nCRT and survival in patients with LACR, which is superior to NLR, PLR, and LMR, and the patients with clinical stage III rectal cancer who have a higher PNI are more likely to benefit from nCRT.

## Introduction

Standard treatment for patients with clinical locally advanced rectal cancer (LARC) includes neoadjuvant chemoradiation therapy (nCRT) followed by total mesorectal excision (TME) and adjuvant chemotherapy (1). This intensive tri-modal therapy is associated with increased local control and sphincter preservation rates and reducing toxicity compared with the postoperative therapy (2). However, individual response to nCRT is variable. Most primary tumors respond well to nCRT, and about 20% of patients even show a pathological complete response (pCR), which may indicate a favorable prognosis (3). Nonetheless, up to one third of patients exhibit resistance to nCRT and the use of nCRT in these patients may result in fatal outcomes because of disease progression or delayed surgery (4). Pathological response to CRT is highly correlated with prognosis in these patients (5). Therefore, there is a need to identify pretreatment factors that can predict the possible therapeutic response and long-term survival, thus aiding in the optimal personalized management of patients with LARC.

Currently, the tumor node metastasis (TNM) staging classification has been recognized as the most powerful prognostic indicator (6). The treatment with or without neoadjuvant therapy in rectal cancer is determined based on the TNM classification (7). Nevertheless, TNM staging classification is far from optimal, because the patients with the same stage tumors may present with different clinical outcomes despite receiving the same standardized treatment (8). Additional markers have been reported with the intention of predicting the prognosis of patients more accurately, including demographic factors such as gender, age, or performance status and clinicopathological tumor-related factors such as carcinoembryonic antigen (CEA) level, perineural invasion, tumor deposits, and circumferential resection margin (9–12). In addition, the treatment outcomes are also driven by host-related factors, especially the pretreatment systemic inflammatory response (SIR) and individual immune-nutritional condition. Various pretreatment biomarkers have been explored. SIR markers such as increased neutrophil to lymphocyte ratio (NLR) and platelet to lymphocyte ratio (PLR) may predict unfavorable prognosis in different types of malignant tumors, meanwhile increased lymphocyte to monocyte ratio (LMR) may be related to better survival outcomes (13–15). The prognostic nutritional index (PNI) is calculated by combining the serum albumin level and lymphocyte count in peripheral blood, and it is an easily measurable index to reflect both nutrition and immune status of the patient (16). Recently, studies also have shown that preoperative PNI is correlated with long-term outcomes, especially for tumors originating from the digestive system (17).

The correlation between SIR and nutrition-immune status can be complex and possibly synergistic for tumor progression. Although previous studies have suggested the potential predictive or prognostic value of these biomarkers, a combined use of SIR markers and immune-nutritional status has never been simultaneously examined in LARC patients as far as we know. Therefore, this study aimed to perform a comprehensive analysis and explore the correlation of pretreatment inflammatory-nutritional biomarkers with responses to nCRT and long-term survival outcomes, thus aiding in the optimal individualized management of patients with LARC.

## Materials and Methods

### Patients

Patients with LARC who underwent nCRT and subsequent TME in our institution between October 2012 and December 2019 were preliminarily screened for this retrospective study. The inclusion criteria were as follows: (1) patient age, 18 to 75 years; (2) pathologically confirmed rectal adenocarcinoma located < 10 cm from anal verge by endoscopic biopsy specimens; (3) radiologically identified clinical staging T3-T4 or lymph node-positive rectal cancer, absence of metastasis, by computed tomography (CT) scan or magnetic resonance imaging (MRI), according to the 7th edition of the American Joint Committee on Cancer-TNM classification; (4) performance status scale of 0–1 according to Eastern Cooperative Oncology Group (ECOG) criteria; (5) no history of prior chemotherapy or pelvic radiotherapy; and (6) complete clinical records, including therapeutic interventions, pathological characteristics of the tumor, and laboratory data within 7 days before nCRT initiation. The exclusion criteria were (1) resections with macro- or microscopically positive pathological margins (R2 or R1); (2) with “watch-and-wait” strategy; (3) with primary malignancies of other organs; (4) with clinical evidence of acute or chronic infection; (5) with hematology or immunology diseases. This study was approved by the Ethics Committee of our institution.

### Data Collection and Definitions

Pretreatment blood biomarkers were calculated from routine laboratory data within 1 week prior to nCRT, including neutrophil, lymphocyte, platelet and monocyte counts, serum albumin, and CEA.

NLR=neutrophil count/lymphocyte count;

PLR= platelet count/lymphocyte count;

LMR=lymphocyte count/monocyte count;

$$\text{PNI}=10\times \text{serum albumin}\;(g/\text{dL})+0.005\times \text{total lymphocyte count}\;(\text{per}\;mm^{3})$$

### Treatment

Patients with LARC in this study underwent nCRT and subsequent TME. Radiotherapy was delivered to the pelvic area with a prescribed dose of 45 Gy in 25 fractions and the primary tumor with a boost dose of 5.4 Gy in three fractions, up to a total dose of 50.4 Gy (18). The intensity-modulated radiation therapy (IMRT) was implemented by using 6-MV Clinac iX linear accelerator (Varian, Palo Alto, CA, USA) in seven to nine equally spaced coplanar fields. Capecitabine was administered at a dose of 825 mg/m^2^ twice daily from Monday to Friday throughout IMRT. One cycle of CapeOX (Oxaliplatin 130 mg/m^2^, day 1, and Capecitabine 1000 mg/m^2^, twice daily, days 1–14) was permitted during the interval from the completion of nCRT to surgery All patients underwent surgery according to the principle of TME at 4 to 8 weeks after nCRT. The postoperative chemotherapy regimen was prescribed as eight cycles of FOLFOX (Oxaliplatin, Leucovorin, and 5-Fluorouracil) or six cycles of CapeOX over approximately 4 months, which was defined as full-dose adjuvant chemotherapy.

### Assessment of Response to nCRT and Follow-Up

Pathological response to nCRT was assessed according to postoperative specimen histopathologic examinations using the tumor regression grade (TRG) system (19). The TRG system was defined as follows: TRG 0 was defined as no remaining viable cancer cells; TRG 1 was defined as single cells or rare residual cancer cells; TRG 2 was defined as residual cancer with a desmoplastic response; TRG 3 was defined as minimal evidence of tumor response. The pCR was defined as TRG 0, and the others were defined as non-pCR. The good-response was defined as TRG 0 and TRG 1, and the poor-response was defined as TRG 2 and TRG 3. Patients were routinely followed up for 5 years according to the following protocol in our institution: 2–4 weeks after discharge, once every 3 months for 1 year, once every 6 months for 2 years, and yearly thereafter. Physical examinations and laboratory tests, including serum CEA levels, were performed at each follow-up visit. The chest and abdominopelvic CT scan was performed every 6 months, and colonoscopy was performed annually or when there was a suspicion of recurrence. Non-routine MRI was performed at the clinician’s discretion. In addition, rigid rectoscopy was performed at each follow-up visit, except when the colonoscopy was performed. Disease-free survival (DFS) was defined as the time from the initiation of nCRT to the development of local recurrence, distant metastasis, or death from any cause (whichever occurred first). Overall survival (OS) was defined as the time from the initiation of nCRT to the date of death or the final follow-up.

### Statistical Analyses

Statistical analyses were performed using the Statistical Package for the Social Sciences, version 23.0 (SPSS Inc., Chicago, IL, USA). Categorical variables were compared using Chi-square test or Fisher’s exact test (if the expected frequencies were <5). Continuous variables were analyzed by using Student’s t-test for normally distributed variables or Mann-Whitney U test for skewed distributed variables. A multivariate logistic regression analysis was performed on statistically significant variables in the univariate analysis using a forward stepwise procedure to examine the final predictors of pathological responses to nCRT. Statistically significant variables in the univariate analysis were further analyzed in the multivariate analysis by using the Cox proportional hazards regression model in a forward stepwise procedure to assess the correlations of predictors with DFS and OS. The X-tile analysis was performed to determine the optimal cutoff value of the statistically significant biomarker to predict total DFS and OS (20). Survival curves were estimated using the Kaplan–Meier method and compared between groups using the log-rank test. The threshold for statistical significance was *P* < 0.05. In univariable Cox regression analyses of DFS and OS, *P* < 0.0026 was considered statistically significant (Bonferroni correction).

## Results

### Patient Characteristics

This study enrolled 356 patients with LARC who underwent nCRT and subsequent TME from October 2012 to December 2019. Patients treated only with neoadjuvant CRT and “watch-and-wait” strategy (n = 14), those who did not complete the course of chemoradiotherapy (n = 4), those who received concurrent oxaliplatin (n = 21) during CRT, those who did not complete the full-dose adjuvant chemotherapy postoperatively (n = 20), those with macroscopically (R2, n = 1) or microscopically (R1, n = 4) positive pathological resection margin, those with incomplete baseline laboratory results (n = 4), those who had no available postoperative histopathology samples (n = 3) and those who were lost to follow-up (n=12) were excluded from this study. Finally, 273 eligible patients were included in this study to maintain homogeneity of the population, especially concerning tumor treatment. Among these patients, 241 (88%) patients underwent pelvic MRI examination for clinical staging assessment, and the other 32 (12%) patients underwent CT scan and endorectal ultrasound to confirm clinical staging because of contraindications to MRI or patients’ willingness. Patient characteristics are summarized in **Table 1**. The median pretreatment levels of NLR, PLR, LMR, and PNI were 3.08 (range, 2.02–6.60), 207.69 (range, 102.31–310.00), 4.33 (range, 2.13–7.00), and 46.00 (range, 36.70–58.55), respectively.

**Table 1:** Patient characteristics and response to nCRT.

Variables	Number (%) (n = 273)	Good response (n = 177)	Poor response (n = 96)	*P*
**Gender**				
Male	177 (64.8%)	116	61	0.742
Female	96 (35.2%)	61	35	
**Age (years)**				
≥60	154 (56.4%)	89	65	**0.006**
<60	119 (43.6%)	88	31	
**ECOG performance status**				
0	131 (48.0%)	91	40	0.124
1	142 (52.0%)	86	56	
**Distance from the anal verge (cm)**				
≥5	153 (56.0%)	104	49	0.220
<5	120 (44.0%)	73	47	
**Pretreatment CEA (µg/L)**				
≥5	139 (50.9%)	70	69	**0.000**
<5	134 (49.1%)	107	27	
**Clinical T stage**				
T1-2	19 (7.0%)	16	3	0.067
T3-4	254 (93.0%)	161	93	
**Clinical N stage**				
N (−)	119 (43.6%)	90	29	**0.001**
N (+)	154 (56.4%)	87	67	
**Chemotherapy during the interval between nCRT and surgery**				
Yes	139 (50.9%)	104	35	**0.000**
No	134 (49.1%)	73	61	
**Pretreatment biomarker levels [median (range)]**				
Neutrophil count	4.39 (1.72–12.88)	4.45 (2.13–12.88)	4.34 (1.72–8.11)	**0.032**
Platelet count	310.15 (130.61–478.58)	310.70 (132.88–478.58)	308.19 (130.61–452.82)	0.815
Lymphocyte count	1.40 (0.60–3.20)	1.50 (0.80–3.20)	1.30 (0.60–2.10)	**0.000**
Monocyte count	0.33 (0.18–0.89)	0.33 (0.19–0.89)	0.33 (0.18–0.58)	0.851
Serum albumin	39.10 (31.20–50.55)	39.40 (31.46–50.55)	38.53 (31.20–49.45)	**0.004**
NLR	3.08 (2.02–6.60)	2.97 (2.02–4.89)	3.20 (2.04–6.60)	**0.025**
PLR	207.69 (102.31–310.00)	197.54 (102.31–304.29)	220.34 (114.55–310.00)	**0.000**
LMR	4.33 (2.13–7.00)	4.58 (2.13–7.00)	3.85 (2.20–6.39)	**0.000**
PNI	46.00 (36.70–58.55)	48.00 (42.12–58.55)	45.25 (36.70–57.60)	**0.000**

nCRT, neoadjuvant chemoradiotherapy; ECOG, Eastern Cooperative Oncology Group; CEA, carcinoembryonic antigen; NLR, neutrophil to lymphocyte ratio; PLR, platelet to lymphocyte ratio; LMR, lymphocyte to monocyte ratio; PNI, prognostic nutritional index. Bold values mean that P-value is significant.

### Correlations Between Pretreatment Biomarkers and Pathological Responses (TRG) to nCRT

Among 273 patients, TRG 0 (pCR) was achieved in 53 (19.4%) patients, TRG 1 in 124 (45.4%), TRG 2 in 50 (18.3%) and TRG 3 in 46 (16.8%), respectively. Totally, 177 (64.8%) patients achieved good-response (TRG 0-1) and 96 (35.2%) patients achieved poor-response (TRG 2-3) to nCRT. The correlations of patient demographic, tumor characteristics, and pretreatment biomarker levels with pathological responses are also available in **Table 1**. In general, higher LMR and PNI were observed in the good-response group, meanwhile higher NLR and PLR were observed in the poor-response group. Multivariate logistic regression analysis results (**Table 2**) revealed that PLR and PNI could independently predict responses to nCRT in patients with LARC.

**Table 2:** Multivariate logistic regression analysis for response to nCRT.

Variables	Hazard Ratio (95% CI)	*P*
**Chemotherapy during the interval between nCRT and surgery**		
Yes	1.837 (1.047–3.224)	**0.034**
No		
**Pretreatment CEA (µg/L)**		
≥5	0.424 (0.236–0.761)	**0.004**
<5		
**Pretreatment biomarkers**		
PLR	0.992 (0.987–0.998)	**0.013**
PNI	1.181 (1.071–1.300)	**0.001**

nCRT, neoadjuvant chemoradiotherapy; CEA, carcinoembryonic antigen; PLR, platelet to lymphocyte ratio; PNI, prognostic nutritional index. Bold values mean that P value is significant.

### Correlations Between Pretreatment Biomarkers and Survival

**Figure 1:**
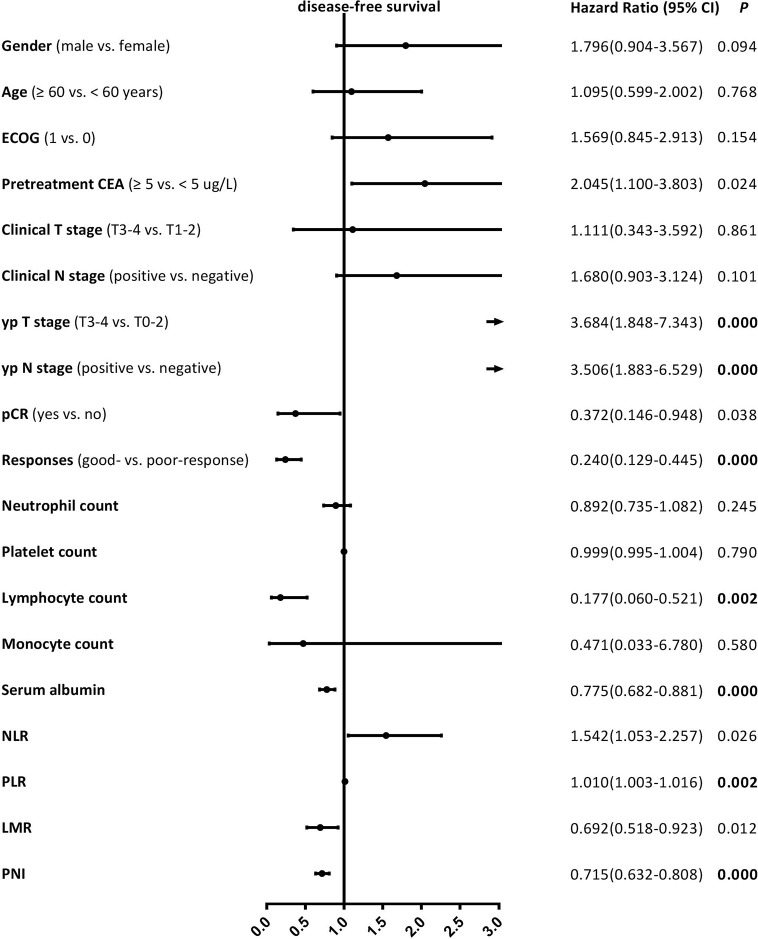
Univariate Cox proportional hazard regression analyses of disease-free survival in patients with locally advanced rectal cancer. Bold value means that P < 0.0026 and it is considered statistically significant after Bonferroni correction.

**Figure 2:**
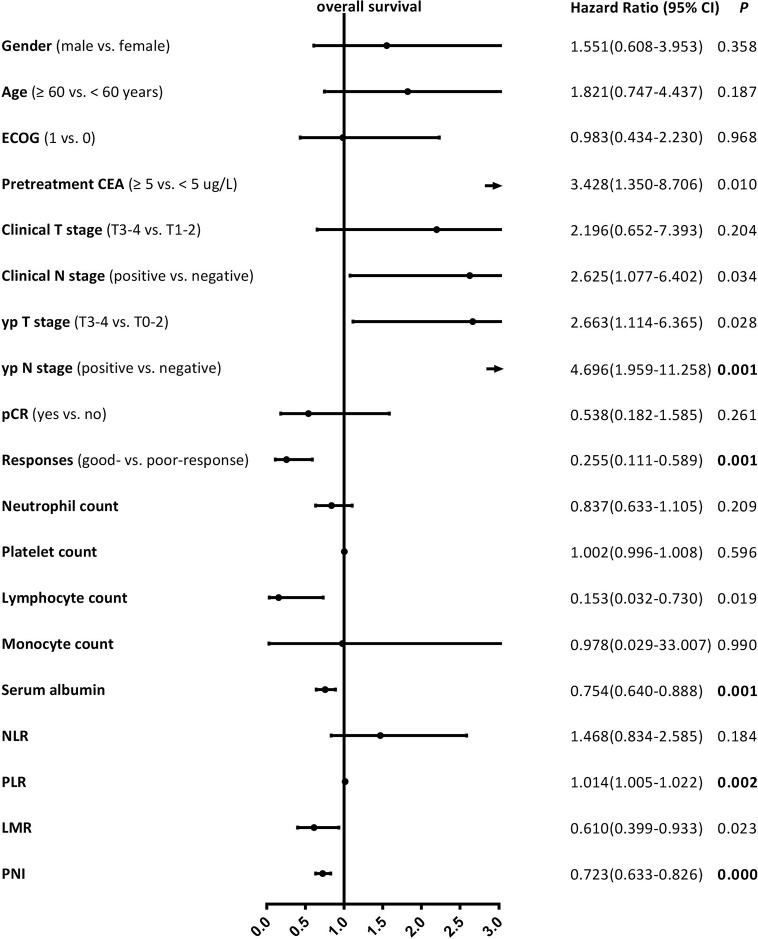
Univariate Cox proportional hazard regression analyses for overall survival in patients with locally advanced rectal cancer. Bold value means that P < 0.0026 and it is considered statistically significant after Bonferroni correction.

Median follow-up time was 42 (range, 10–78) months, while median DFS was 38 (range, 10–78) months. The 5-year DFS and OS rates were 73.1% and 78.9% for the entire cohort, respectively. The patients with good-response had a significantly better 5-year DFS (81.2% vs. 58.5%, ***P* = 0.000**) and OS (83.6% vs. 70.9%, ***P* = 0.001**) rates compared with those with poor-response. The univariate analysis results revealed that the lymphocyte count, serum albumin, PLR, and PNI were significantly correlated with DFS, as well as yp T stage, yp N stage, and pathological responses (**Figure 1**), and the serum albumin, PLR, and PNI were significantly correlated with OS, as well as yp N stage and pathological responses (**Figure 2**). Multivariate Cox regression analysis determined that pathological responses and PNI were independent predictors of DFS, and yp N stage and pretreatment PNI were independent predictors of OS in patients with LARC (**Table 3**).

**Table 3:** Multivariate Cox proportional hazard regression analyses of DFS and OS.

Variables	DFS		OS	
	Hazard Ratio (95% CI)	*P*	Hazard Ratio (95% CI)	*P*
**Pathological responses**				
Good-response vs. poor-response	0.357 (0.188–0.678)	**0.002**	–	–
**yp N stage**				
Positive vs. negative	–		2.880 (1.118–7.422)	**0.028**
**Pretreatment biomarkers**				
PNI	0.750 (0.663–0.849)	**0.000**	0.767 (0.672–0.876)	**0.000**

DFS, disease-free survival; OS, overall survival; NLR, neutrophil to lymphocyte ratio; PNI, prognostic nutritional index. Bold values mean that P value is significant.

### Subgroup Analysis to Assess the Clinical Utility of the Pretreatment PNI in Predicting Pathological Responses to nCRT and Survival

**Figure 3:**
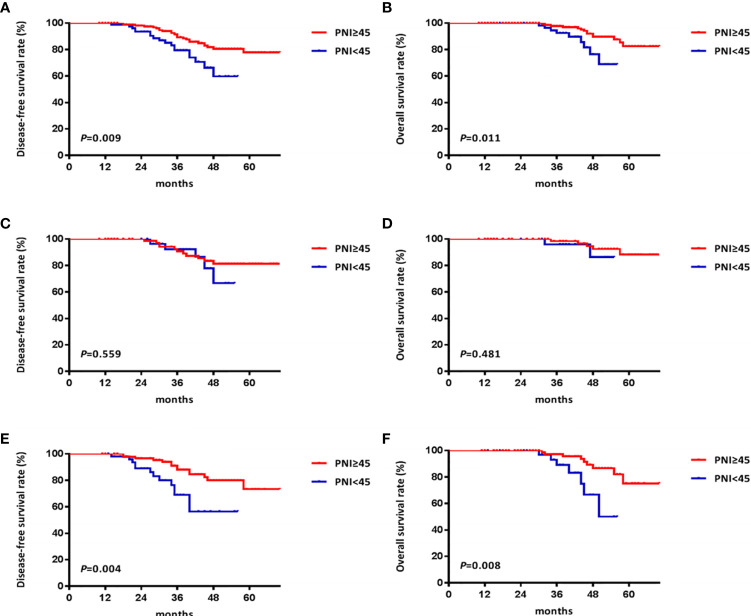
Kaplan Meier survival curves of patients with locally advanced rectal cancer grouped by prognostic nutritional index (PNI) and stratified by clinical stage. **(A)** the disease-free survival (DFS) curves of all patients; **(B)** the overall survival (OS) curves of all patients; **(C)** the DFS curves of patients with clinical stage II rectal cancer; **(D)** the OS curves of patients with clinical stage II rectal cancer; **(E)** the DFS curves of patients with clinical stage III rectal cancer; **(F)** the OS curves of patients with clinical stage III rectal cancer.

X-tile analysis determined that the optimal cutoff value of the PNI was 44.9 for total DFS and 44.8 for total OS. Considering that the optimized cutoff value of PNI was 45 in the initial pivotal study of Onodera et al. (21), we determined the PNI cutoff value as 45 in the present study. Patients were dichotomized into high PNI group [PNI ≥ 45; n = 177 (64.8%)] and low PNI group [PNI < 45; n = 96 (35.2%)]. The good-response rate in the high PNI group was significantly higher than that in the low PNI group (69.5% vs. 56.3%, ***P*=0.029**). The DFS and OS rates in the high PNI group were also significantly better than those in the low PNI group (5-year DFS: 77.9% vs. 59.7%, ***P*=0.009**, **Figure 3A**; 5-year OS: 82.5% vs. 68.8%, ***P*=0.011**, **Figure 3B**).

We further analyzed the utility of PNI in predicting pathological responses to nCRT and survival based on the clinical TNM stage in patients with LARC. Among patients with clinical stage II rectal cancer (n = 119), higher good-response rate and better survival outcomes were observed in the high PNI group compared with the low PNI group (good-response rate: 77.5% vs. 71.8%, *P* = 0.496; 5-year DFS: 81.3% vs. 66.7%, *P* = 0.559, **Figure 3C**; 5-year OS: 88.3% vs. 86.4%, *P* = 0.481, **Figure 3D**), but the differences were not statistically significant. Among patients with clinical stage III rectal cancer (n = 154), comparable results were observed between the high and low PNI groups, and the differences were statistically significant (good-response rate: 62.9% vs. 45.6%, ***P* = 0.037**; 5-year DFS: 73.4% vs. 56.5%, ***P* = 0.004**, **Figure 3E**; 5-year OS: 75.2% vs. 49.9%, ***P* = 0.008**, **Figure 3F**).

## Discussion

To the best of our knowledge, this is the first time to evaluate the predictive and prognostic values of these four pretreatment inflammatory and nutritional factors for LARC in one study. The findings suggested that a higher pretreatment PNI is correlated with better pathological responses and prognosis in patients with LARC undergoing nCRT, superior to the established SIR markers such as NLR, PLR, and LMR. Moreover, pretreatment PNI can be used as a supplemental tool in predicting responses to nCRT and survival based on TNM classification for LARC.

It is increasingly recognized that the initiation and progressions of rectal cancer are not solely determined by the inherent characteristics of the tumor but also by host-related factors (22). There may be substantial cross-talk between the systemic inflammation and immune responses against cancer cells and the surrounding microenvironment, and the interaction mechanism is far from being fully understood (23). Although the independent utility of the NLR, PLR, LMR, and PNI as predictors of responses to treatment or patient prognosis have achieved promising results in the published literature, including rectal cancer (24–27), the results have often been controversial when these biomarkers are evaluated simultaneously in the same patient cohort (28). What is more, previous studies usually attempted to identify factors correlated with response to neoadjuvant therapy or long-term survival as two separate entities, the persistence forecasting abilities of these biomarkers remain unknown. Therefore, this study was conducted to investigate the correlations of a range of biomarkers with not only responses to nCRT but also long-term outcomes such as DFS and OS in patients with LARC.

TRG is recommended as the preferred grading method of rectal cancer response to treatment by the AJCC Staging Manual and the College of American Pathologists Guidelines (29). Therefore, TRG was selected to assess the correlation between these biomarkers and the responses to nCRT in this study. Furthermore, although tumor pathological response to nCRT is considered to be correlated with prognosis, the final endpoint should still be long-term outcomes to evaluate the predictive value of these biomarkers (30). Previous literature achieved mixed results regarding the correlation of SIR markers with tumor response or prognosis. Kim et al. reported that NLR, LMR, and PLR could not be used to distinguish total tumor regression from the residual disease after nCRT; while higher PLR was correlated with improved recurrence-free survival (31). Michael et al. found that NLR and PLR are neither independent predictors of response nor prognostic factors in LARC patients undergoing nCRT followed by radical surgery (32). However, the data from William et al. showed that baseline lower levels of LMR and higher levels of NLR and PLR were correlated with decreased OS (25). The superior indicator value of PNI on the prognosis of patients with LARC has been validated, but there are limited studies on the correlation between PNI and responses (17, 33). In this study, PNI showed a better, more consistent, and independent predictive ability for treatment response and prognosis in univariate and multivariate analyses, other SIR markers such as NLR, PLR, and LMR did not show an independent predictive ability in multivariate analysis, which may be related to the inherent correlation between such indicators and PNI, and this phenomenon has been described in some studies (34, 35), but it still needs to be further explored.

Serum albumin is a broadly recognized indicator for nutrition status, and initial studies documented that the albumin can be used to assess disease progression and prognosis (36, 37). However, there is increasing evidence to suggest that the prognostic value of albumin may be subordinate to an ongoing SIR, so the albumin should be used in combination with other markers to enhance prognostic value (38, 39). Lymphocytes play a crucial role in the host immune response to cancer, which is associated with improved outcomes in solid tumors according to previous reports (40). The results of this study showed that the lymphocytes and serum albumin were significantly correlated with responses to nCRT and survivals in univariate analysis but not in multivariate analysis. PNI is composed of serum albumin level and peripheral lymphocyte count, and it may reflect both the nutritional and immunological status of a patient (21). The multivariate analysis in this study determined that the PNI was an independent predictor of response and prognosis in patients with LARC undergoing nCRT.

Several explanations could contribute to the correlation of PNI with treatment response and prognosis in patients with LARC. Firstly, Capecitabine, the oral fluorinated pyrimidine prodrug, is recommended concomitantly with radiotherapy as a radiation sensitizer. Capecitabine is readily absorbed in the gastrointestinal tract and it requires the presence of thymidine phosphorylase for its conversion into the active form of 5-fluorouracil within the cells (41). Low albumin concentrations may result in abnormal pharmacokinetics and inferior bioavailability, and continuing inflammation at baseline also had significantly decreased metabolic activities of chemotherapy drugs and increased their toxicities, thus leading to unfavorable response and clinical outcomes (42). What is more, LARC patients often develop malnutrition as a result of insufficient food intake, malabsorption, and increased metabolic demands. It has been proposed that malnutrition is related to cytokine-driven inflammation and may lead to the immunosuppressed condition, which can be reflected by hypoalbuminemia and low lymphocyte counts (43). This immunosuppressed condition might be responsible for the insufficient anti-tumor immune response and provide an advantageous microenvironment for tumor progression in low-PNI patients (44). Additionally, patients with decreased PNI may have an enhanced SIR (45). The excessive inflammatory components can further lead to increased depletion of fat stocks, as well as protein degradation in skeletal muscle and other host tissue. The absence of albumin can lead to immune regulation dysfunction by affecting the stabilized cell growth, DNA replication, and antioxygenation *in vivo*, and the albumin degradation products may serve as source of nutrient substrates for accelerating tumor growth and proliferation (46). Inflammation is also an important regulator of tumor progression through suppression of albumin synthesis, recruitment of T lymphocytes and tumor-associated macrophages, and upregulation of angiogenic growth factors (47). The precise mechanisms underlying the correlation between PNI and treatment outcomes may be complex and our understanding of this process remains unclear. Nevertheless, the potential predictive and prognostic values of PNI exist in providing an inexpensive, non-invasive and effective supplemental tool.

Of note, this study differed from previous studies in methodology. A common feature of previous studies was to dichotomize continuous biomarkers primitively to stratify patients into different subgroups to explore the correlations of these biomarkers with response or prognosis, and thus the cut off values of these biomarkers are various in different literature (17, 24–28, 31–35). In this study, we choose not to dichotomize these biomarkers primitively, because the forecasts of continuous variables have enormous advantages from a statistical standpoint. This allowed us to minimize false-positive results and establish a much more accurate forecast model by using Cox proportional hazard model. Our findings suggested that increased PNI was correlated with higher good-response and better long-term outcomes across the continuous range of PNI. If this finding is further confirmed and thus PNI can be considered as a routine test during the first clinical visit, interventions to improve PNI will produce an additive benefit on treatment outcomes in LARC patients, not just those with a PNI above a predefined cut off value. Interestingly, the predictive and prognostic values of pre-treatment PNI were greater in patients with clinical stage III rectal cancer. This finding could be mainly attributed to the fact that the patients with clinical stage III rectal cancer were more likely to suffer from high tumor burden and long-term nutritional consumption, which might up-regulate the expressions of cytokines and inflammatory mediators, leading to immunosuppressed host and decreased local immune response, thus affecting the sensitivity of nCRT and the long-term survival (23).

Currently, available data recommends nCRT followed by TME as the standard treatment for patients with LARC. However, not all LARC patients respond to nCRT, which subgroup population would benefit from nCRT remains unclear. In the present study, we observed the differences in response to nCRT and survival between high and low PNI groups. Patients with a higher PNI are more likely to benefit from nCRT. These findings indicate that the baseline inferior immunonutritional status may impair the efficacy of nCRT. At present, the prospective clinical evidence of immunonutritional intervention during oncological treatment remains limited and our present knowledge about these is still at a rudimentary stage. However, there may exist potential therapeutic target that can alter the disease course (48). Therefore, it is necessary to pay more attention to the assessment of the immunonutritional status, to provide better guidance for clinical treatment, especially in patients with clinical stage III rectal cancer, who might need additional supportive interventions to further improve their prognoses. In this context, the use of PNI as a surrogate marker of inherent immunological status in a host can provide a new perspective on optimizing strategies for individualized management of patients with LARC.

Following prior studies, we found that chemotherapy during the interval between nCRT and surgery and the pretreatment CEA were independently correlated with responses to nCRT in patients with LARC (9, 49, 50). We also found that responses to nCRT and yp N stage were independent predictors of DFS and OS, respectively (4, 35, 51, 52). In addition, the 5-year DFS and OS rates in the present study are similar to those previously reported in several landmark trials of nCRT in patients with LARC (2, 53, 54). These findings confirm that the current cohort is truly representative of patients with LACR undergoing nCRT and thus support the validity and generalizability of our results.

There are several limitations in this study. The retrospective nature of this type of analysis is subject to shortcomings such as potential data collection and selection bias. This was a single-center and single-race study and the optimal cutoff values for these biomarkers may fluctuate in other heterogenous patient cohorts. Additionally, the PNI is a non-specific biomarker that can be affected by various pathophysiologic conditions and thus will vary from time to time. In this study, we mainly focused on the correlation between baseline inflammatory-nutritional factors and clinical outcomes to aid in the optimal individualized management of patients with LARC. However, the impact of changes in these markers over time has yet to be determined. Therefore, further studies are required to confirm the results of this current study. Finally, C-reactive protein (CRP) was not a preoperative routine examination in our center, so the predictive value of CRP or CRP-based indicator such as Glasgow prognostic score (55) was not analyzed in this study. However, the lack of available CRP data reminds us that currently inflammatory marker detection has not entered clinical practice and it needs to be further explored in the future.

## Conclusion

In summary, this study confirmed that PNI can serve as a promising predictor of response to nCRT and survival in patients with LACR, and patients with a higher PNI are more likely to benefit from nCRT, especially for patients with clinical stage III rectal cancer. Whilst these results are required to be re-validated in prospective trials, PNI routinely collected before treatment may assist in better risk-stratifying patients and thus aid in the determination of an optimal individual treatment plan for a patient with LARC.

## Data Availability Statement

The original contributions presented in the study are included in the article/**Supplementary Material**. Further inquiries can be directed to the corresponding author.

## Ethics Statement

The studies involving human participants were reviewed and approved by the Ethics Committee of Jiangsu Province Hospital of Chinese Medicine. The patients/participants provided their written informed consent to participate in this study.

## Author Contributions

DY: study design and guidance, critical revision of the manuscript, and study supervision. YW: data acquisition and analysis, statistical evaluation of the results, and drafting of the manuscript. LC, BZ, WS, and LX: data acquisition, analysis, and interpretation. GZ: software application. All authors contributed to the article and approved the submitted version.

## Funding

This work was partly supported by the Traditional Chinese Medicine Science and Technology Planning Project of Jiangsu Province (No. JD201801).

## Conflict of Interest

The authors declare that the research was conducted in the absence of any commercial or financial relationships that could be construed as a potential conflict of interest.
