# Supplementary material for: Pretreatment Inflammatory-Nutritional Biomarkers Predict Responses to Neoadjuvant Chemoradiotherapy and Survival in Locally Advanced Rectal Cancer
Source: Front Oncol. 2021 Mar 17;11:639909. doi: 10.3389/fonc.2021.639909 (PMC8010250; doi:10.3389/fonc.2021.639909)
Supplement: Supplementary file 1 [file DataSheet_1.doc]

Supplementary Material

## Supplementary Figures


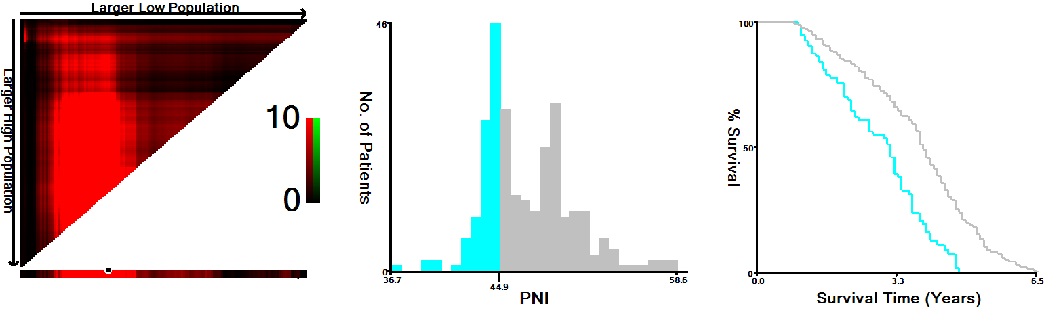


**Figure S1** **|** X-tile analyses to determine the optimal cut-off values of PNI for the DFS. The optimal cut-off values of PNI was 44.9.


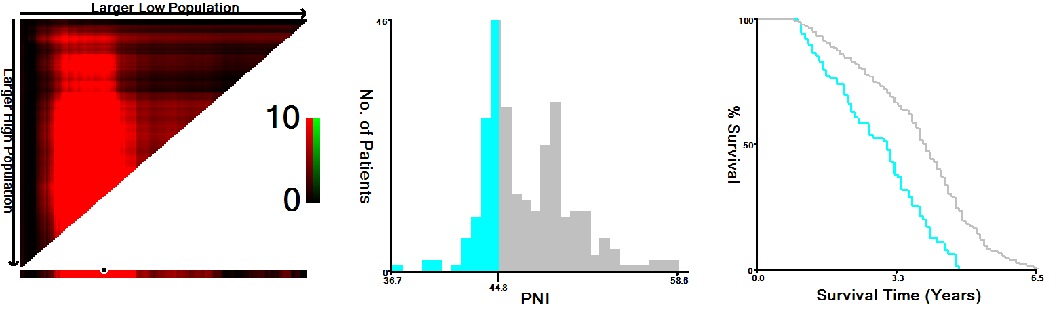


**Figure S2** **|** X-tile analyses to determine the optimal cut-off values of PNI for the OS. The optimal cut-off values of PNI was 44.8.
